# Adolescent Overweight and Obesity: Links to Socioeconomic Status and Fruit and Vegetable Intakes

**DOI:** 10.3390/ijerph13030307

**Published:** 2016-03-09

**Authors:** Jihyun You, Jina Choo

**Affiliations:** College of Nursing, Korea University, Anam-Dong, Seongbuk-Gu, Seoul 137-705, Korea; jhemin@korea.ac.kr

**Keywords:** social class, fruit and vegetable intake, adolescent, obesity, overweight

## Abstract

Whether adolescent overweight/obesity is linked to socioeconomic status (SES) and fruit and vegetable (F/V) intakes has not been confirmed. We aimed to determine whether there is an association between SES and adolescent overweight/obesity and to test the mediating effect of F/V intakes. This cross-sectional study included the data of 63,111 adolescents extracted from the 2013 Korea Youth Risk Behavior Web-based Survey. Overweight/obesity was defined as a body mass index ≥ 85th percentile, while F/V intakes were categorized as high (recommended levels: ≥1 fruit serving and ≥3 vegetable servings per day) *versus* low. Among girls, low SES (beta = 0.50, *p* < 0.001) and F/V intakes (beta = −0.17, *p* = 0.038) were both significantly associated with overweight/obesity; the former association was significantly mediated by F/V intakes (Sobel test: z = 2.00, *p* = 0.046). Among boys, neither SES nor F/V intakes was significantly associated with overweight/obesity. Adolescent overweight/obesity was significantly linked to low SES and F/V intakes among girls only; low SES indirectly increased the risk of overweight/obesity via low F/V intakes. Therefore, promoting F/V intakes for socially disadvantaged girls should be prioritized as a population-based strategy for preventing adolescent overweight/obesity in South Korea.

## 1. Introduction

Adolescent overweight/obesity has emerged as a major public health concern, as its prevalence has increased remarkably across countries of all economic level [1]. In the United States, the prevalence of adolescent overweight/obesity (body mass index (BMI) ≥ 85th percentile) increased from 30.4% to 34.5% between 2000 and 2012 [2,3]. In South Korea, the prevalence of obesity (BMI ≥ 95th percentile) increased from 9.2% to 12.7% between 1998 and 2013 [4].

Adolescent overweight/obesity increases the risk of adult overweight/obesity [5,6]. Specifically, a previous study reported that adolescent obesity at 10–17 years of age was more likely to be associated with the risk for adulthood obesity than childhood obesity at 1–9 years of age [7]. Moreover, adolescent overweight/obesity is reportedly associated with an increased risk of metabolic syndrome in young adulthood and increased risks of cardiovascular disease and cancer in later adulthood [8,9,10]. Therefore, identifying determinants associated with adolescent overweight/obesity is necessary for preventing its adverse consequences such as latent obesity and morbidity in adulthood.

Socioeconomic status (SES) may be a determinant of adolescent overweight/obesity [11]. A recent systematic review reported that SES and child-adolescent weight status were inversely associated in rich countries and that its magnitude increased with age [12]. Several previous studies, mostly in Europe and the U.S., also revealed that inverse associations between SES and overweight/obesity significantly differed by gender in childhood and adolescence [13,14], showing a stronger association in girls than in boys [13] or a significant association in girls only [14]. Such an inverse association in girls has been speculated due to unmeasured behavioral factors associated with SES [14]. 

Fruit and vegetable (F/V) intakes are healthy eating behaviors that may be best fostered in childhood and adolescence based on their proven significant health benefits of preventing diabetes mellitus, cardiovascular disease, and some cancers [15,16,17]. Reportedly, low F/V intakes are a precursor of overweight/obesity [18], while high F/V intakes have beneficial effects on preventing excessive weight gain in adulthood [19,20]. Low F/V intakes are reportedly associated with low SES, particularly shown more prevalent in socially disadvantaged adolescent girls than those boys [21]. However, so far, evidence about the links between F/V intakes and overweight/obesity in adolescence with the concomitant consideration of SES is scarce. 

We thus posited that low SES and low F/V intakes would be linked to adolescent overweight/obesity and tested whether low F/V intakes would mediate the association between low SES and overweight/obesity in Korean adolescent boys and girls. Specifically, four hypotheses were proposed for the present study: (1) low SES would be significantly associated with overweight/obesity in adolescent boys and girls; (2) low SES would be significantly associated with low F/V intakes in adolescent boys and girls; (3) low F/V intakes would be significantly associated with overweight/obesity in adolescent boys and girls after adjusting for SES; and (4) low F/V intakes would mediate the association between low SES and overweight/obesity in adolescent boys and girls. To investigate these hypotheses, we used nationally representative data of a Korean adolescent population obtained from the 2013 Korea Youth Risk Behavior Web-based Survey (KYRBWS) [22].

## 2. Materials and Methods 

### 2.1. Design and Participants

This cross-sectional, population-based study included the data of 63,111 boys and girls that were extracted from the 2013 KYRBWS [22]. The KYRBWS is officially approved by Statistics Korea (certificate number 11758) and is repeated annually by the Korea Centers for Disease Control and Prevention; Ministry of Health and Welfare; and Ministry of Education, Science, and Technology to investigate health-related behaviors of Korean adolescents aged 12–18 years including smoking, alcohol drinking, physical activity, eating behaviors, and weight status [23]. KYRBWS data are collected using a multistage, stratified, cluster-sampling method that involves stratification, clustering, weight, and finite population correction. The population was stratified according to regional and school-type variables, and the sample was then clustered and selected according to school and class [22]. In the KYRBWS, Korean adolescents in grades 7–12 grades at middle and high schools voluntarily and anonymously complete a web-based self-administered questionnaire. Written informed consent was received from all participants and their parents or legal guardians. The online self-reported survey was performed in school computer rooms after the participants were fully informed about the survey and assigned identification numbers to ensure anonymity. The total number of participants in the 2013 KYRBWS was 72,435 boys and girls. After excluding those with missing data, a total of 63,111 boys and girls were included in the final analysis.

### 2.2. Measurements

For the present study, sociodemographic, behavioral, and anthropometric characteristics were extracted from the 2013 KYRBWS data. Sociodemographic characteristics included age, school type (middle or high), learning achievement (low or high), residential setting (city or rural), living arrangement (with or without family), parents’ nationality (Korean or foreign), adolescents’ perceived SES (high, mid-high, mid, mid-low, or low), and parents’ highest education level (categorical: ≤9, 10–12, or ≥13 years). The parents’ highest education level was defined as the highest of either the father’s or mother’s education levels. We combined and recoded the two indicators of adolescents’ perceived SES and parents’ highest education levels to create a SES variable of low *versus* high SES: perceived SES was recoded as high (high, mid-high, mid SES) *versus* low (mid-low, low SES), and parents’ highest education levels were recoded as high (≥13 years, college-educated) *versus* low (<13 years, less college-educated). In the present study, low SES was defined as both low perceived SES and low parents’ highest education, whereas high SES was defined as any other combination. Previously, the SES indicators of adolescents’ perceived SES [24,25] and parents’ highest education levels [12,21,26,27] were reported to associate significantly with the health status, health behaviors, and overweight/obesity among adolescents. Moreover, these two SES indicators exhibited a significant correlation with each other (Spearman rho = 0.3, *p* < 0.001). In this regard, a combined SES variable involving these two SES indicators might be more robust than each of the indicators alone with respect to associations with F/V intakes and overweight/obesity among adolescents; in fact, the combined SES variable was found to be more robust (data not shown).

The behavioral characteristics used in the present study were F/V intakes, fast-food intakes, regular exercise, and sedentary behavior. F/V intakes were measured using two questions: “How often did you eat fruit (not including fruit juice) during the past 7 days?” and “How often did you eat vegetables (not including kimchi) during the past 7 days?”, and recoded as high (recommended level: ≥1 fruit serving and ≥3 vegetable servings per day) *versus* low (below the recommended level) according to the criteria of the Dietary Guidelines for Adolescents from the Korea Ministry of Health and Welfare [28,29,30]. Fast food intakes were measured by asking the participants, “How often did you eat fast foods such as fried chicken, hamburgers, or pizza during the recent 7 days?” and recoded as ever-consuming (≥1 time during the recent 7 days) *versus* never-consuming [31].

Regular exercise was measured by asking participants, “How often did you exercise moderately and vigorously during the past 7 days?” and recoded as high (recommended level: moderate physical activity every day and vigorous physical activity ≥3 days per week) *versus* low (below the recommended level) according to the criteria of the 2008 Physical Activity Guidelines for Americans from the Centers for Disease Control and Prevention [32,33]. Sedentary behavior was measured by asking, “How many hours did you sit during the recent weekdays?” and recoded as low (recommended level: <2 h per day of sedentary time during the recent weekday) *versus* high (below the recommended level) [33]. All of the recommended levels were defined according to the criteria of the Korea Ministry of Health and Welfare, U.S. Centers for Disease Control and Prevention, and Canada Center for Disease Control and Prevention [28,31,32,33].

Anthropometric characteristics were obtained as self-reported body weight (kg) and height (cm). BMI was calculated as the weight in kilograms divided by the square of the height in meters (kg/m^2^), and categorized as non-overweight/obese (BMI < 85th percentile) *versus* overweight/obese (BMI ≥ 85th percentile) using sex- and age-specific BMI percentiles based on the Korea Centers for Disease Control and Prevention growth charts [34].

### 2.3. Ethical Considerations

The study was approved by the Institutional Review Board at Korea University (KU-IRB-15-EX-254-A-1) and all procedures were followed in accordance with the ethical standards of this board.

### 2.4. Data Analysis

Participants (*n* = 63,111, weighted n (wn) = 3,272,722) were included in the final analysis and analyzed by gender-stratified group using the complex samples procedure. “wn” refers to the population size determined by multiplying the values of weight (w) and sample size (n). All statistical analyses were performed using SPSS 23.0 (SPSS Inc., Chicago, IL, USA).

The participants’ general characteristics are expressed as either weighted means (standard errors) or numbers (weighted %) as appropriate. To test differences between boys and girls in the participants’ sociodemographic, behavioral, and anthropometric characteristics, an independent *t*-test or chi-square test was used as appropriate. Logistic regressions were performed to examine crude associations of sociodemographic and behavioral characteristics with overweight/obesity and independent associations of SES and F/V intakes with overweight/obesity after adjusting for covariates. We included the following covariates in adjusted regression models based on the results of crude associations with overweight/obesity (*p* < 0.2): age, school type, residential setting, learning achievement, parents’ nationality, fast-food intake, regular exercise, and sedentary behavior.

Finally, to test four hypotheses, including the mediating effect of F/V intakes on the association between SES and adolescent overweight/obesity, three equations were examined via logistic regressions [35]. To test Hypothesis 1 (SES would be significantly associated with overweight/obesity in adolescent boys and girls), overweight/obesity (outcome) was regressed on SES (predictor) in the first equation. To test Hypothesis 2 (SES would be significantly associated with F/V intakes in adolescent boys and girls), F/V intakes (outcome) were regressed on SES (predictor) in the second equation. To test Hypothesis 3 (F/V intakes would be significantly associated with overweight/obesity in adolescent boys and girls after adjustment for SES), overweight/obesity (outcome) was regressed on F/V intakes after adjusting for SES in the third equation. To test Hypothesis 4 (F/V intakes would significantly mediate the association between SES and overweight/obesity in adolescent boys and girls), the Sobel test was used to evaluate the potential mediation effect of F/V intakes on the association between SES and overweight/obesity, with regression coefficients and standard errors obtained from the results of Hypotheses 2 and 3. The Sobel test is used to test the significance of a mediation effect by determining whether the reduction in the effect of the predictor after including the mediator in the model is significant and, therefore, whether the mediation effect is statistically significant [36]. Values of *p* < 0.05 were considered statistically significant.

## 3. Results

### 3.1. Participants’ General Characteristics

The participants (*n* = 63,111, wn = 3,272,722) had a mean age of 14.8 years (range, 12–18 years); of them, 49.7% were girls and 46.5% were high school students (Table 1). Of the total participants, 10.8% lived in a rural setting. Girls were significantly less likely to be overweight and obese than boys (13.4% of girls versus 15.2% of boys with a BMI ≥ 85th percentile, *p* < 0.001). Girls were significantly more likely to have low F/V intakes (*p* = 0.004) and low regular exercise (*p* < 0.001), and high sedentary behavior than boys (*p* < 0.001). Moreover, girls were more likely than boys to be of low SES (*p* = 0.026).

### 3.2. Crude Associations of Sociodemographic and Behavioral Characteristics with Overweight/Obesity

Girls who were younger (*p* < 0.001), enrolled in high schools (*p* = 0.045), reported themselves as learning less-achieved (*p* < 0.001), had low F/V intakes (*p* = 0.026), and never-consumed fast-foods in the recent 7 days (*p* < 0.001) were significantly more likely than their counterparts to be overweight or obese (Table 2). Boys who reported themselves as learning less-achieved (*p* < 0.001), who never-consumed fast-food intakes (*p* < 0.001) during the recent 7 days, and had low regular exercise during the recent 7 days (*p* < 0.001) were significantly more likely than their counterparts to be overweight or obese. 

### 3.3. Testing of Four Hypotheses: Associations among SES, F/V Intakes, and Overweight/Obesity 

Among girls, low SES was significantly and positively associated with overweight/obesity in the first equation (Hypothesis 1) (B = 0.509, *p* < 0.001), and significantly and inversely associated with F/V intakes in the second equation (Hypothesis 2) (B = −0.835, *p* < 0.001) (Table 3). In the third equation, F/V intakes (B = −0.171, *p* < 0.038) were significantly and inversely associated with overweight/obesity after adjusting for SES (Hypothesis 3). Moreover, a low SES continued to exert a significant direct influence on overweight/obesity (B = 0.505, *p* < 0.001), although the magnitude of this association slightly decreased after adjusting for F/V intakes in the third equation (Hypothesis 4). 

**Figure 1:**
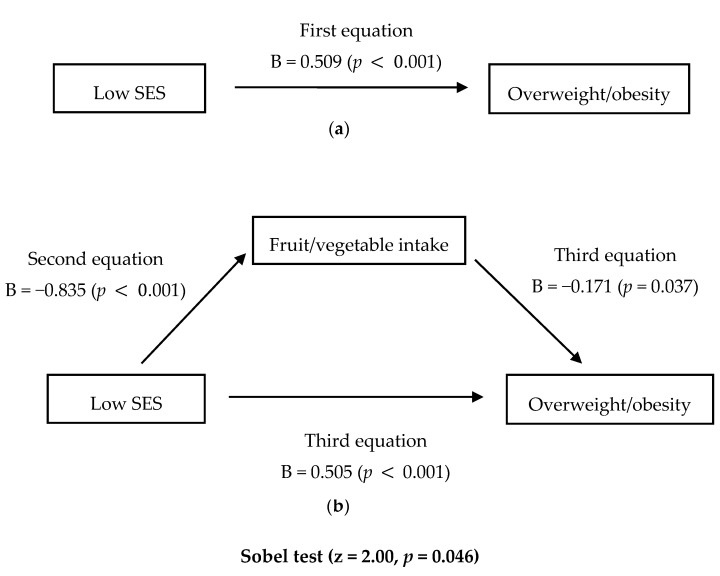
Adolescent overweight/obesity linked to socioeconomic status and fruit and vegetable intakes in adolescent girls: Testing for a mediating effect of fruit/vegetable intakes on the association between SES and overweight/obesity using a logistic regression analysis (**a**) Direct pathway from low SES to overweight/obesity; (**b**) Indirect pathway from low SES to overweight/obesity via fruit/vegetable intakes. B: standardized regression coefficient

Hence, F/V intakes had a partial mediating effect on the association between low SES and overweight/obesity among girls (Hypothesis 4) (Sobel test; z = 2.00, *p* = 0.046) (Figure 1). However, among boys, low SES was not significantly associated with overweight/obesity in the first equation (Hypothesis 1), but was significantly and inversely associated with F/V intakes in the second equation (Hypothesis 2) (B = −0.765, *p* < 0.001). In the third equation, F/V intakes was not significantly associated with overweight/obesity with adjustment for SES (Hypothesis 3). Based on this, we did not conduct a Sobel test for testing Hypothesis 4.

## 4. Discussion

In this Korean adolescent population, low SES was significantly associated with overweight/obesity, while low F/V intakes had a significant mediating effect on the association between low SES and overweight/obesity. However, the links between low SES, F/V intakes, and adolescent overweight/obesity differed by gender; among girls, low SES and low F/V intakes were significantly associated with overweight/obesity and low SES was significantly associated with overweight/obesity via low F/V intakes. Among boys, neither low SES nor F/V intakes was significantly associated with overweight/obesity.

We found that low SES was significantly associated with overweight/obesity among adolescent girls but not adolescent boys; in fact, gender significantly modified the association between SES and overweight/obesity in a pooled data of adolescent boys and girls (*p* < 0.001; data not shown). This gender-specific association between SES and adolescent overweight/obesity was consistent with the findings of several previous studies [12,37,38], although all studies were not consistent. The Health Behavior in School-aged Children Study, a WHO collaborative cross-national study [39] reported that family affluence, a SES indicator, was inversely associated with overweight/obesity in both boys and girls in approximately half of 39 included countries. However, Singh *et al*. [13] examined the association of neighborhood SES and built environment with overweight/obesity among 91,642 children aged 10–17 years in the USA and found that the impacts of neighborhood SES and built environment on obesity (not on overweight) were significantly stronger for girls than for boys. Furthermore, Zahnd *et al*. [14] examined the association between socioeconomic disadvantage and obesity among 2648 elementary school students in the USA, and found that socioeconomically disadvantaged girls were at a higher risk of obesity than were non-socioeconomically disadvantaged girls; however, such an association was not seen among boys. Such a gender-specific association was attributed to socioeconomically disadvantaged girls having low access to healthy food and physical environments, which, in turn, predisposed them to increased vulnerability to overweight/obesity. 

As stated above, our findings demonstrate that the significant association between low SES and adolescent overweight/obesity was partially and significantly mediated by low F/V intakes among adolescent girls. Meanwhile, the non-significant association between low SES and overweight/obesity among adolescent boys in the present study may have been moderated in part by other factors. Specifically, the Korean patriarchal culture [40] may play a role in protecting the effects of low SES on the risk for overweight/obesity, not though low F/V intakes. Traditionally, boys may be prioritized to be served other healthy foods (in contrast, they may also be leniently permitted fast foods that can be purchased by their own allowance) and signed up in activity facilities despite the family being socially disadvantaged, whereas girls may be marginalized instead. Furthermore, son preference value embedded in Asian countries such as Korea and China may explain girls’ low access to healthy behaviors. Wang [41] reported that boys in a family were more likely to be highly educated than girls based on the belief that boys provide financial benefits for their families. In this context, we speculate that low educated girls may be less informed of and have fewer healthy lifestyle skills, specifically Asian country context. However, these speculations might not be generalizable to non-Asian country context. 

Meanwhile, we also found that low F/V intakes were significantly associated with overweight/obesity among adolescent girls but not boys. Fruit and vegetables are an important source of fiber and contain high amounts of water and low amounts of saturated fat, which are related to reduced energy density, hunger control, and satiety [42]. Heo *et al*. [20] reported an inverse association between F/V intakes and overweight/obesity among 430,912 adults using the 2007 Behavioral Risk Factor Surveillance Survey in the U.S. Whigham *et al*. [43] examined the effects of high F/V intakes on long-term weight loss using the measure of serum carotenoids, called as a biomarker of F/V intakes, and reported that high F/V intakes significantly correlated with weight loss for obese adults. Further, this empirical evidence allowed intervention studies to include a high F/V diet as a weight management strategy [42]. However, few studies investigated the association between F/V intakes and overweight/obesity among adolescents. In this reason, we could not discuss a gender-specific association between low F/V intakes and overweight/obesity based on the previous literature. However, previous studies elucidated that genetic, behavioral, and parental factors influencing adolescent overweight/obesity differed by gender [44]. In our data, activity behaviors, measured by regular exercise, rather than eating behaviors, measured by F/V intakes, were inversely and significantly associated with overweight/obesity among boys (B = −0.23, *p* = 0.001 for boys; data not shown), but not among girls. In this context, different behavioral factors may influence the increased risk of overweight/obesity in girls and boys.

To the best of our knowledge, this is the first study to elucidate the mediating effect of F/V intakes on the association between SES and overweight/obesity in a representative adolescent population. Moreover, this study distinguishes itself from previous studies by reporting data after the full adjustment for potential confounding factors (*i.e.*, age, residential setting, learning achievement, fast food intakes, regular exercise, and sedentary behavior) that were, in previous studies, identified in the association between SES and adolescent overweight/obesity [45,46]. 

The present study had limitations. First, its cross-sectional nature may not guarantee causal inferences about the mediating effect of F/V intakes on the association between SES and overweight/obesity. Therefore, longitudinal research is needed to understand the causal relationships. Second, the self-reported anthropometric data obtained from the 2013 KYRBWS may underestimate (or overestimate) the actual prevalence of overweight/obesity [47,48]. Furthermore, F/V intake data may require a concomitant assessment with additional complementary measures of a food frequency questionnaire or 24-hour dietary recall. Third, our results may not be generalizable to other ethnic populations or low- and middle-income countries. 

## 5. Conclusions 

Adolescent overweight/obesity was significantly linked to low SES and F/V intakes. Low SES, specifically via low F/V intakes, may indirectly increase the risk for adolescent overweight/obesity in girls but not boys. Therefore, promoting F/V intakes for socially disadvantaged girls should be prioritized as a population-based strategy for preventing adolescent overweight/obesity in South Korea.

## Figures and Tables

**Table 1:** Participants’ general characteristics (*n* = 63,111), wn = 3,272,722.

	n (Weighted %), Mean (SE)						t/χ^2^	*p*
	All		Girls		Boys			
			(*n* = 31,343)		(*n* = 31,768)			
**Sociodemographic Characteristics**								
Age	14.8	(0.05)	14.9	(0.06)	14.7	(0.07)	1.54	<0.001
School type							7.24	0.518
Middle	31,894	(53.5)	16,102	(54.0)	15,792	(52.9)		
High	31,217	(46.5)	15,241	(46.0)	15,976	(47.1)		
Learning achievement							151.85	<0.001
High	7057	(11.5)	3086	(9.9)	3971	(13.0)		
Low	56,054	(88.5)	28,257	(90.1)	27,797	(87.0)		
Residental setting							74.67	0.166
City	55,717	(89.2)	27,925	(90.3)	27,792	(88.2)		
Rural	7394	(10.8)	3418	(9.7)	3976	(11.8)		
Living arrangement							30.47	0.023
With family	60,607	(96.3)	30,201	(96.7)	30,406	(95.9)		
Without family	2504	(3.7)	1142	(3.3)	1362	(4.1)		
Parents’ nationality							0.001	0.976
Korean	62,460	(99.0)	31,010	(99.0)	31,450	(99.0)		
Foreign	651	(1.0)	333	(1.0)	318	(1.0)		
SES							16.90	0.026
High	55,685	(88.6)	27,520	(88.1)	28,165	(89.1)		
Low	7426	(11.4)	3826	(11.9)	3603	(10.9)		
**Behavioral characteristics**								
F/V intakes (per day) ^1^							11.61	0.004
Low	59,695	(94.5)	29,728	(94.8)	29,967	(94.2)		
High	3416	(5.5)	1615	(5.2)	1801	(5.8)		
Fast-food intakes							4.09	0.146
Ever-consuming	43,530	(68.9)	21,484	(68.5)	22,046	(69.2)		
Never-consuming	19,581	(31.1)	9859	(31.5)	9722	(30.8)		
Regular exercise ^2^							1095.37	<0.001
Low	60,212	(95.3)	30,763	(98.1)	29,449	(92.5)		
High	2899	(4.7)	580	(1.9)	2319	(7.5)		
Sedentary behavior ^3^							250.57	<0.001
High	38,207	(60.8)	19,982	(63.8)	18,225	(57.7)		
Low	24,904	(39.2)	11,3611	(36.2)	13,543	(42.3)		
**Anthropometric characteristics**								
BMI							43.52	<0.001
Non-overweight/obese	54,076	(85.7)	27,145	(86.6)	26,931	(84.8)		
Overweight/obese	9035	(14.3)	4198	(13.4)	4837	(15.2)		

BMI: body mass index; F/V: fruit/vegetable; SE: standard error; SES: socioeconomic status; ^1^ F/V intake (high): >1 fruit serving and >3 vegetable servings per day; ^2^ Regular exercise (high): Moderate physical activity every day and vigorous physical activity 3 days per week; ^3^ Sedentary behavior (high): >2 hours per day of sedentary time during the recent weekday.

**Table 2:** Crude associations of sociodemographic and behavioral characteristics with overweight/obesity among adolescents (*n* = 63,111), wn = 3,272,722.

Characteristics	Girls (*n* = 31,343)			Boys (*n* = 31,768)		
	OR	(CI)	*p*	OR	(CI)	*p*
**Sociodemographic Characteristics**						
Age (ref: younger)	0.84	(0.78–0.91)	<0.001	1.07	(1.00–1.15)	0.065
School type (ref: middle schools)	1.08	(1.00–1.17)	0.045	1.06	(0.98–1.14)	0.158
Residential setting (ref: city)	1.10	(1.00–1.22)	0.066	0.95	(0.83–1.09)	0.467
Living arrangement (ref: with family)	0.98	(0.84–1.15)	0.810	0.91	(0.77–1.08)	0.282
Learning achievement (ref: high)	1.38	(1.23–1.54)	<0.001	1.22	(1.11–1.35)	<0.001
Parents’ nationality (ref: Korean)	1.11	(0.83–1.49)	0.496	0.76	(0.54–1.07)	0.115
SES (ref: high)	1.66	(1.52–1.80)	<0.001	1.13	(1.02–1.25)	0.020
**Behavioral Characteristic**						
F/V intakes (ref: low) ^1^	0.84	(0.71–0.98)	0.026	0.93	(0.81–1.05)	0.233
Fast-food intakes (ref: never-consuming)	0.76	(0.72–0.82)	<0.001	0.86	(0.80–0.92)	<0.001
Regular exercise (ref: low) ^2^	1.03	(0.81–1.31)	0.798	0.79	(0.69–0.90)	<0.001
Sedentary behavior (ref: low) ^3^	1.06	(1.00–1.13)	0.071	1.04	(0.97–1.12)	0.295

CI: confidence interval; F/V: fruit/vegetable; OR: odds ratio; ref: reference group; SES: socioeconomic status; ^1^ F/V intake: ≥1 fruit serving and ≥3 vegetable servings per day; ^2^ Regular exercise: Moderate physical activity every day and vigorous physical activity 3 days per week; ^3^ Sedentary behavior: ≥2 h per day of sedentary time during the recent weekday.

**Table 3:** Testing for a mediating effect of fruit/vegetable intakes on the association between SES and overweight/obesity among adolescents (*n* = 63,111), wn = 3,272,722.

Variables	Girls ^1^			Boys ^1^		
	B	SE	*p*	B	SE	*p*
**First equation**						
Outcome: overweight/obesity						
Predictor: low SES	0.509	0.042	<0.001	0.093	0.052	0.076
**Second equation**						
Outcome: F/V intake						
Predictor: low SES	−0.835	0.113	<0.001	−0.765	0.119	<0.001
**Third equation**						
Outcome: overweight /obesity						
Mediator: F/V intake	−0.171	0.082	0.038	−0.040	0.068	0.554
Predictor: low SES	0.505	0.042	<0.001	0.092	0.053	0.082

B: standardized regression coefficient; SE: standard error; ^1^ The logistic regression models adjusted for age, school type, residential setting, learning achievement, parents’ nationality, fast-food intake, regular exercise, and sedentary behavior.
